# Identification and analysis of a prognostic ferroptosis and iron-metabolism signature for esophageal squamous cell carcinoma

**DOI:** 10.7150/jca.68568

**Published:** 2022-03-06

**Authors:** Mengnan Zhao, Ming Li, Yuansheng Zheng, Zhengyang Hu, Jiaqi Liang, Guoshu Bi, Yunyi Bian, Qihai Sui, Cheng Zhan, Miao Lin, Qun Wang

**Affiliations:** Department of Thoracic Surgery, Zhongshan Hospital, Fudan University, Shanghai 200032, China.

**Keywords:** esophageal squamous cell carcinoma, GEO database, TCGA database, TMB, GDSC database, Connectivity Map database

## Abstract

**Background:** The role of ferroptosis in esophageal squamous cell carcinoma (ESCC) is still unclear.

**Methods:** The association of iron metabolism and ferroptosis-related genes with the prognosis, copy number variation (CNV), TMB, and immune cell infiltration of ESCC was explored using data from the GEO and TCGA database and validated by immunofluorescence in 112 ESCC patients from our center. The potential anti-cancer drugs and compounds from the GDSC and the Connectivity Map database were also screened.

**Results:** A total of 117 iron metabolism and ferroptosis-related genes were identified. We found the expressions of PRNP, SLC3A2, SLC39A8, and SLC39A14 negatively related to the prognosis of ESCC patients, while ATP6V0A1 and LCN2 were opposite, which was validated in 112 ESCC samples from our center. And a prognostic signature was constructed based on their expressions and Cox regression coefficient (β). The low-score group exhibited a significantly worse OS. Besides, analysis of 179 ESCC samples from GSE53625 revealed that patients of poorly differentiation, more than 60 years, T4 stage, advanced N stage, advanced stage, and adjuvant therapy also exhibited a significantly shorter OS, based on which a nomogram to predict the OS was established. Moreover, the low-score group exhibited significantly higher CNV and TMB and more frequent mutations of TP53, MUC16, and NOTCH1. Higher proportion of Macrophages M2, and lower proportion of T cells follicular helper were observed in the low-score group. We discovered that AZD7762, Sunitinib, Cytarabine, Docetaxel, Vinblastine, and Elesclomol exhibited lower IC50 in the low-score group. And 20 potential compounds were identified from the CMap database.

**Conclusions:** Six iron metabolism and ferroptosis-related genes were associated with the prognosis, CNV, TMB, and immune cell infiltration of ESCC. Some potential anti-cancer drugs and compounds may be helpful for OS.

## Introduction

The esophagus is responsible for transporting food from the pharynx to the stomach. Esophageal cancer, often leading to stenosis, greatly affects the quality of patients' life with an estimated 450,000 deaths per year and quite low 5-year-survival-rate which is less than 20% worldwide 1. It can be principally classified into two different histological subtypes: esophageal squamous cell carcinoma (ESCC) and esophageal adenocarcinoma (EAC), exhibiting distinct epidemiological features and risk factors. ESCC accounts for approximately 90% of all esophageal cancer cases, especially in the East Asia and the Middle East, with tobacco smoking and alcohol use as risk factors. Whereas, EAC is prevalent in developed countries with acid or bile reflux and Barrett esophagus as risk factors 2. Most ESCC patients were initially diagnosed at advanced stage, who require adjunctive therapy to prolong their survival. Therefore, it is an urgent to discover novel biomarkers to predict prognosis, radiosensitivity, and drug sensitivity of ESCC patients.

As an iron-dependent form of nonapoptotic cell death, ferroptosis was first proposed by Dixon et al in 2012, induced by peroxidation of phospholipids 3. Many articles revealed the tumor suppression and immune surveillance role of ferroptosis. It has been demonstrated that the inhibition of SLC7A11 by p53 resulted in the reduction of glutathione (GSH), which promoted the ferroptosis and tumor suppression both *in vitro* and *in vivo* 4-6. Besides, Wang et al have reported that interferon-γ secreted by CD8+ T cells enhanced the sensitivity of tumor cells to ferroptosis through the suppression of SLC3A2 and SLC7A11 7. The ferroptosis-inducing therapies have drawn much attention. The combinations of ferroptosis-based treatment with radiotherapy, immunotherapy or other therapies have also been explored 6. Lang et al reported that radiotherapy and immunotherapy could promote ferroptosis and tumor suppression through synergistic repression of SLC7A11 8. Lei et al also demonstrated that radiotherapy provoked ferroptosis in esophageal cancer with a better disease-free survival 9.

In this study, we explored the association of iron metabolism and ferroptosis-related genes with the prognosis, copy number variation (CNV), TMB, and immune cell infiltration of ESCC using data from the Gene Expression Omnibus (GEO) database and The Cancer Genome Atlas (TCGA) database and validated it in ESCC samples from our center. In addition, we also screened potential anti-cancer drugs and compounds from the Genomics of Drug Sensitivity in Cancer (GDSC) database and the Connectivity Map (CMap) database. Our results may improve the survival of ESCC patients with personalized treatment.

## Patients and Methods

### Ethics Statement

This study was approved by the Ethics Committee of Zhongshan Hospital, Fudan University, Shanghai, China (Approval No. B2017-153). Written informed consent was obtained from all patients.

### Identification of iron metabolism and ferroptosis-related genes

Ferroptosis-related genes (map04216) were identified from the KEGG PATHWAY Database (https://www.genome.jp/kegg/pathway.html). Iron metabolism-related genes were identified from cellular iron ion homeostasis (GO: 0006879) of the AmiGo2 database (http://amigo.geneontology.org/amigo) and the pathway of iron uptake and transport (R-HSA-917937) from the Reactome Pathway Database (https://reactome.org/).

### Identifying differentially expressed iron metabolism and ferroptosis-related genes (DEGs) between ESCC and normal esophageal tissues

The expression data of ESCC and normal esophageal tissues were extracted from the GSE20347, GSE67269, and GSE38129 datasets. To better eliminate the batch effect in different researches, all 120 pairs of samples were tested by the GPL571 chip. The DEGs were obtained using “limma” R package with the threshold of absolute log (fold change) > 1 and adjusted P value < 0.05. In addition, the RNA-Sequencing profile and clinical information of 96 ESCC patients were collected from the TCGA database to investigate the relation of DEGs to the prognosis, TMB, immune cell infiltration, CNV, and drug sensitivity.

### Construction and functional analysis of iron metabolism and ferroptosis-related prognostic signature

The relation of the expression of DEGs to the prognosis of ESCC patients was analyzed with multivariate Cox regression, based on which a prognostic signature was constructed. The prognostic score was the sum of prognostic genetic expressions times their Cox regression coefficient (β). Prognostic score = (β * expression level of PRNP) + (β * expression level of SLC3A2) + (β * expression level of SLC39A8) + (β * expression level of SLC39A14) + (β * expression level of ATP6V0A1) + (β * expression level of LCN2). The optimal cut-off value of the prognostic score was determined by the "survminer" R package, based on which the patients were divided into low- and high-score group 10. The predictive power of the gene signature was evaluated by the time-dependent receptor operating characteristic (ROC) curves.

The Gene Ontology (GO) and Kyoto Encyclopedia of Genes and Genomes (KEGG) analysis were performed to explore the biological function using Broad Institute's GSEA software 4.1 (https://www.gsea-msigdb.org/gsea/index.jsp) 11.

### Cell culture and siRNA transfection

Two ESCC cell lines (KYSE150 and ECA109) were purchased from the Chinese Academy of Science Cell Bank and cultured with Dulbecco's Modified Eagle's Medium (Hyclone, UT, USA), supplemented with 10% fetal bovine serum (Every Green, Hangzhou, China) and 100 IU/mL penicillin/streptomycin (Beyotime, Shanghai, China) in a humidified 5% CO_2_ atmosphere at 37 °C.

The expressions of the above 6 prognostic genes were knock-down with 2 diiferent small interfering RNAs (siRNAs) (RiboBio, Guangzhou, China) (Table S1). The siRNAs were transfected into cells with Lipo8000 transfect reagent (Beyotime, Shanghai, China) and Opti-MEM (Thermo Fisher Scientific, MA, USA) according to the manufacturer's protocol.

### RNA extraction and quantitative real-time polymerase chain reaction

Total RNA of cultured cells was extracted using RNAiso Plus (Takara Biomedical Technology, 9108) according to the manufacturer's protocol. cDNA templates were synthesized using PrimeScript™ RT reagent Kit with gDNA Eraser (Takara Biomedical Technology, RR047A). Subsequently, qRT-PCR was performed using TB Green® Premix Ex Taq ™ II (Takara Biomedical Technology, RR820A) in an Applied Biosystems system 7500 (Thermo Fisher Scientific, Waltham, MA USA) according to the manufacturer's protocol. Primer sequence were listed in Table S1.

### Lipid peroxidation assay

The lipid peroxidation of treated cells were measured as previously reported 9. Briefly, the cells were stained with 10uM BODIPY 581/591 C11 dye (Invitrogen, D3861) before flow cytometry in an Accuri 6 cytometer (BD, Bioscience).

### Prognostic validation of the ferroptosis-related prognostic genes

To validate the prognostic accuracy of the above signature, 179 ESCC samples from the GSE53625 were analyzed. We also built a nomogram to predict overall survival (OS). The predictive power of the nomogram was assessed by the Harrel concordance index (C-index) and comparing the nomogram-predicted and observed Kaplan-Meier estimates of survival probability 12.

We validated the prognostic role of the above 6 genes in 112 ESCC patients from our center. Patients received neoadjuvant therapy were excluded. The relative expressions of the above 6 genes were measured by immunofluorescence as previously described. Briefly, the paraffin-embedded slides were dewaxed and rehydrated. After antigen retrieval, block of endogenous peroxidase activity and non-specific antigens, and incubation with primary antibodies: anti-PRNP (Affinity Biosciences LTD, DF7034), anti-SLC3A2 (Affinity Biosciences LTD, DF7468), anti-SLC39A14 (Affinity Biosciences LTD, DF14224), anti-SLC39A8 (Affinity Biosciences LTD, DF7743), anti-ATP6V0A1 (Abcam, ab204737), and anti-LCN2 (Affinity Biosciences LTD, DF6816) and horseradish peroxidase-conjugated secondary antibody, the slides were incubated with Opal tyramide signal amplification (TSA) Fuorochromes (Opal 2-Color Manual IHC Kit, G1226, Servicebio Co., Ltd) for 10 min at room temperature. After the second run, the slides were stained with DAPI. The fluorescence intensity was analyzed using imageJ software 13. The association of their expressions and prognosis was evaluated by Kaplan-Meier analysis.

### Characteristics of CNV, TMB, and immune cell infiltration in two groups

The “maftools” R package was used to analyze TMB, defined as the number of somatic mutations per megabase of interrogated genomic sequence 14-15. The CNV was identified with the GISTIC algorithm 16. The CIBERSORT method was applied to evaluate the relative abundance of the 22 immune cell subpopulations based on the transcriptome data 17. Correlations of key gene expression and immune cell infiltration were analyzed in the TIMER database.

### Analysis of potential anti-cancer drugs and compounds

The Genomics of Drug Sensitivity in Cancer (GDSC) database was searched to discover potential anti-cancer drugs, which describes the responses to 138 anti-cancer drugs across 1000 cancer cell lines 18. Besides, we screened the potential activated or inhibited compounds based on gene expression signatures in the Connectivity Map (CMap) database, which contains 42,080 genetic and small-molecule perturbations profiled from numerous human cell lines 19.

## Results

### Identification of prognostic ferroptosis-related DEGs between ESCC and normal esophageal tissues

From the KEGG PATHWAY Database, the AmiGo2 database, and the Reactome Pathway Database, a total of 117 ferroptosis-related genes were identified (Table S2). Among them, only 22 ferroptosis-related DEGs which fulfilled the inclusion criteria (absolute log FC > 1 and adjusted P value < 0.05) were enrolled based on the RNA-sequencing profile of 120 pairs of ESCC and normal esophageal tissues from the GSE20347, GSE67269, and GSE38129 datasets (Figure S1). Unfortunately, the three datasets did not provide survival information. Thus, we collected the RNA-Sequencing profile and clinical information of 96 ESCC patients from the TCGA database.

**Figure 1:**
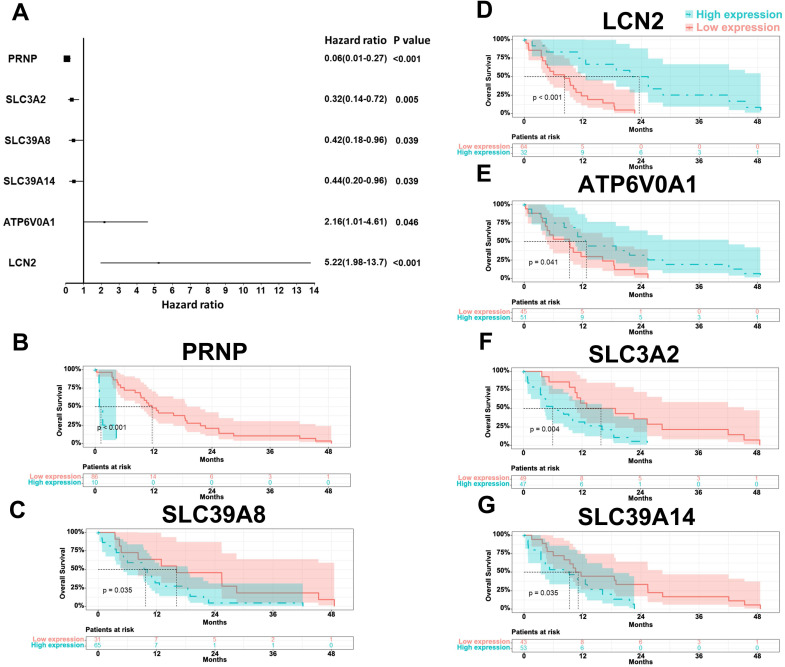
Forest map (A) and Kaplan-Meier curves of PRNP (B), SLC39A8 (C), LCN2 (D), ATP6V0A1 (E), SLC3A2 (F), and SLC39A14 (G) based on the TCGA database.

From the TCGA database, out of the 22 ferroptosis-related DEGs, the expressions of 6 genes were significantly associated with prognosis after multivariate Cox regression. As shown in Figure 1, the expressions of PRNP (p < 0.001), SLC3A2 (p = 0.005), SLC39A8 (p = 0.039), and SLC39A14 (p = 0.039) negatively related to the prognosis, while ATP6V0A1 (p = 0.046) and LCN2 (p < 0.001) were opposite.

**Figure 2:**
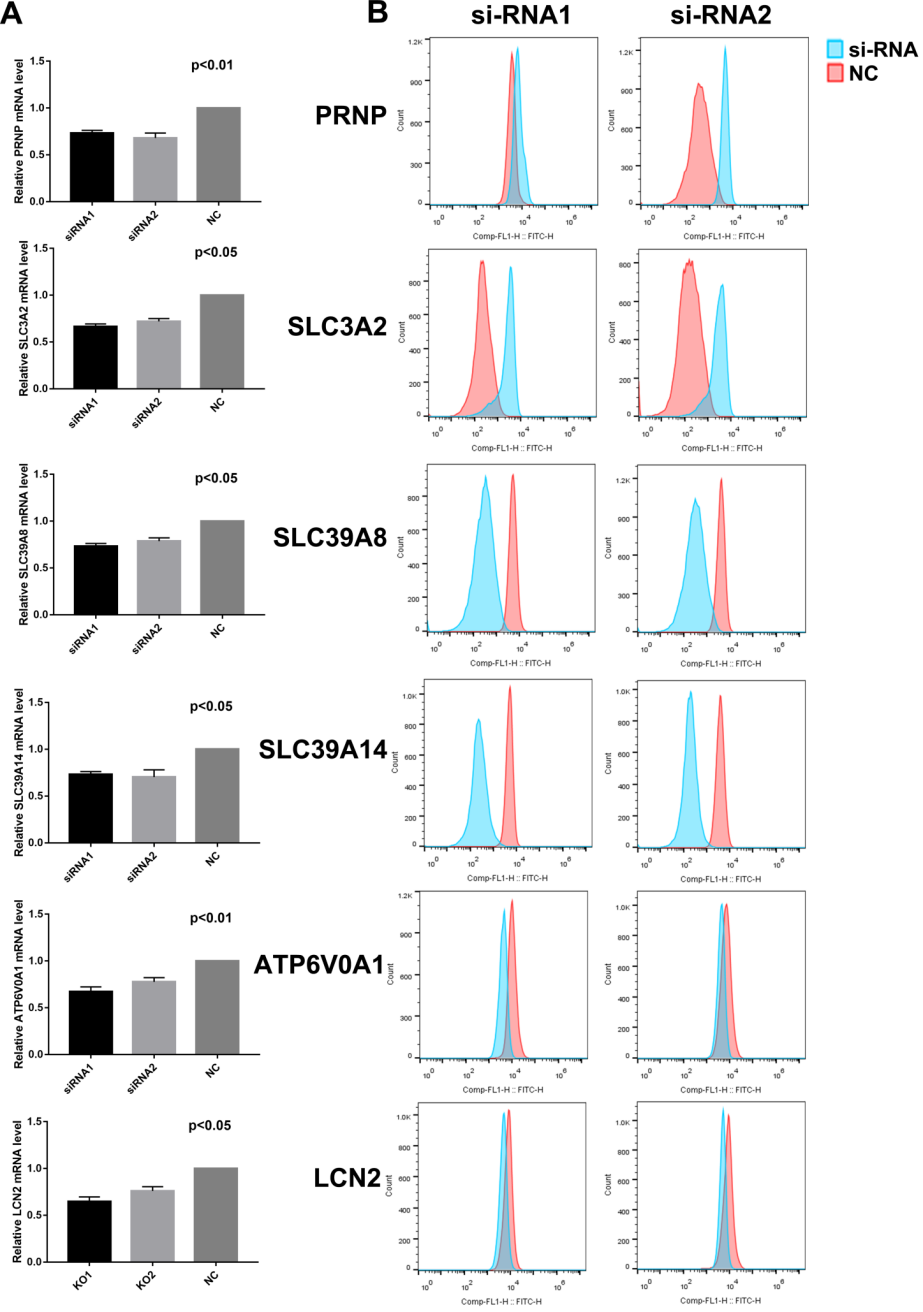
Relative mRNA expressions (A) and lipid peroxidation (B) after transfection with siRNAs in KYSE150 cells.

Subsequently, we analyzed the effects of these 6 genes on ferroptosis of ESCC cell lines. After transfection with 2 different siRNAs, ferroptosis was inhibited in SLC39A8, SLC39A14, ATP6V0A1, and LCN2 knock-down cells, which was opposite in PRNP and SLC3A2 knock-down cells (Figure 2, Figure S2).

### Construction and functional analysis of prognostic signature for ESCC patients

**Figure 3:**
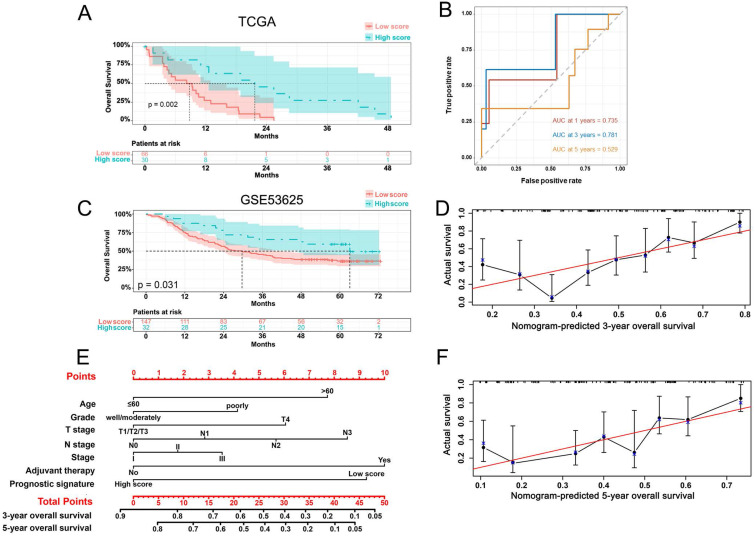
Kaplan-Meier curve (A) and AUC of time-dependent ROC curves (B) of ESCC patients in the low and high score group based on the TCGA database and Kaplan-Meier curve (C), nomogram (E) and calibration plots (D, F) of ESCC patients from the GSE53625 dataset.

A prognostic signature for ESCC patients was constructed based on the expressions of previous 6 prognostic genes and their Cox regression coefficient (β). Prognostic score = (0.06 * expression level of PRNP) + (0.32 * expression level of SLC3A2) + (0.42 * expression level of SLC39A8) + (0.44 * expression level of SLC39A14) + (2.16 * expression level of ATP6V0A1) + (5.22 * expression level of LCN2). Then, we used the “survminer” R package to calculate the optimal cut-off score, based on which the 96 ESCC patients were divided into high- and low-score group. The low-score group exhibited a significantly worse OS (p = 0.002) (Figure 3A). The area under the curve (AUC) in the time-dependent ROC at 1, 3 and 5 years reached 0.735, 0.781, and 0.529 (Figure 3B).

GSEA analysis revealed that the retromer complex, spindle midzone, and regulation of protein autophosphorylation gene sets were enriched in the low-score group (Figure S3A). As for the KEGG pathway analysis, the low-score group was associated with adherens junction, focal adhesion, ECM receptor interaction, cell cycle, DNA replication, and homologous recombination, which relate to cell proliferation and migration. Interestingly, the gene sets of renal cell carcinoma, colorectal cancer, prostate cancer, and small cell lung cancer were enriched in the low-score group (Figure S3B).

### Validation of the prognostic signature and establishment of a nomogram for overall survival

To validate the accuracy of prognostic signature, expression data and clinical information from 179 ESCC samples (GSE53625) were analyzed. As shown in Figure 3C, the low-score group had a significantly worse OS (p = 0.031). The 5-year overall survival was 36.6 percent in the low-score group and 59.4 percent in the high-score group. Besides, patients of poorly differentiation, more than 60 years, T4 stage, advanced N stage, advanced stage, and adjuvant therapy also exhibited a significantly shorter OS (Figure S4). We established a nomogram to predict the OS based on these prognosis-related parameters. To estimate the 3-year and 5-year OS, add the points of each parameter together based on the points scale at the top and obtain the OS rates based on the total points scale at the bottom (Figure 3E). The bootstrap re-sampling calibration plot demonstrated a good consistency between the predicted and actual OS, which was also proven by the C-index (0.682, 95% CI = 0.629-0.734), suggesting the good predictions of the nomogram (Figure 3D, 3F).

**Figure 4:**
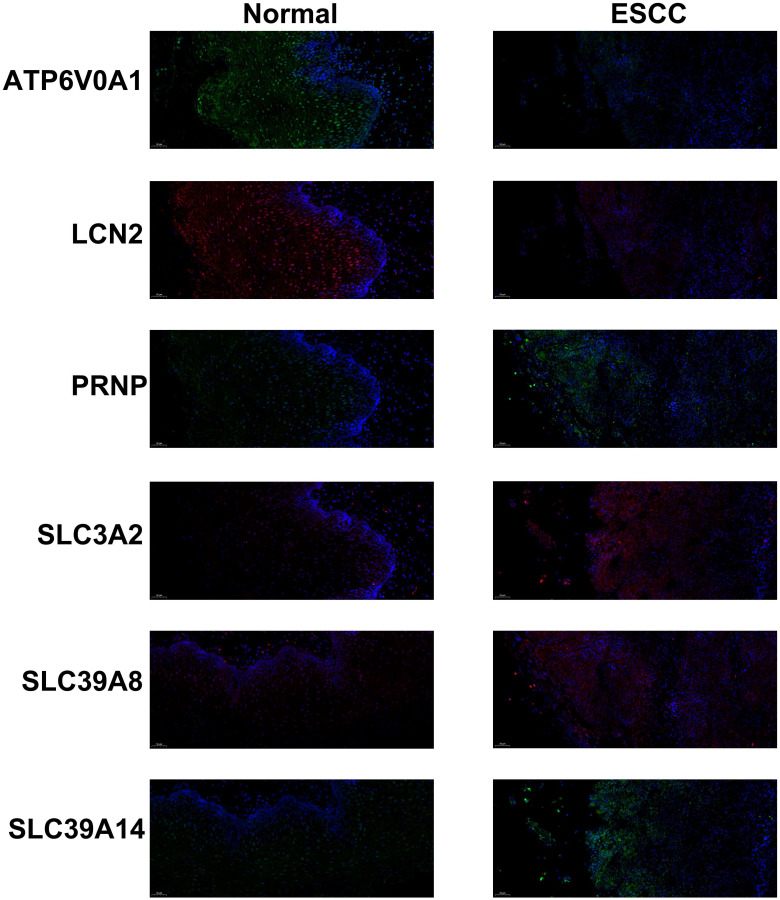
Immunofluorescence of PRNP (green), SLC39A8 (red), LCN2 (red), ATP6V0A1 (green), SLC3A2 (red), and SLC39A14 (green) in ESCC samples and normal tissues.

Moreover, the expressions of the 6 prognostic genes were also assessed by immunofluorescence in 112 ESCC patients from our center (Table 1). Similarly, we observed lower expressions of ATP6V0A1 and LCN2 and higher expressions of PRNP, SLC3A2, SLC39A14, and SLC39A8 in the ESCC samples, compared to the normal tissues (Figure 4). Furthermore, the expressions of ATP6V0A1 (p = 0.043) and LCN2 (p = 0.025) were favorable for OS. On the contrary, the expressions of PRNP (p = 0.031), SLC3A2 (p = 0.006), and SLC39A14 (p = 0.047) were adverse. Notably, the expression of SLC39A8 (p = 0.035) was unfavorable, which was different from the above results and large-scale studied were needed to clarify its accurate prognostic role (Figure S5).

### Landscape of genetic variations and immune cell infiltration

**Figure 5:**
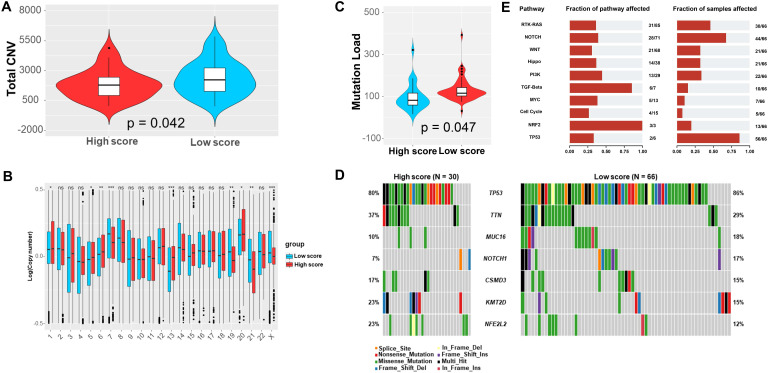
Total CNV (A), distribution of CNVs in each chromosome (B), TMB (C), and most common mutations of genes (D) and pathways (E) in the low and high score group based on the TCGA database.

Totally, the low-score group exhibited significantly more CNV (Figure 5A). Specifically, the CNV of chromosome 7, 19, 21, and the X were significantly more frequent in the low-score group, which were converse for chromosome 1, 5, 6, 13, and 20 (Figure 5B). Furthermore, we observed that the low-score group had greatly higher TMB (Figure 5C). Notably, there were more frequent mutations of TP53, MUC16, and NOTCH1 in the low-score group (Figure 5D). Correspondingly, the dysfunction of RTK-RAS, NOTCH, and TP53 pathway were more common in the low-score group (Figure 5E). Moreover, MUC17 was significantly likely to co-mutated with DNAH5, KMT2D, and KMT2A. Interestingly, TP53 was significantly mutually exclusive with KMT2D in the low score group. However, MUC17 was significantly likely to co-mutated with BRD4 and CACNA1C in the high score group (Figure S6).

**Figure 6:**
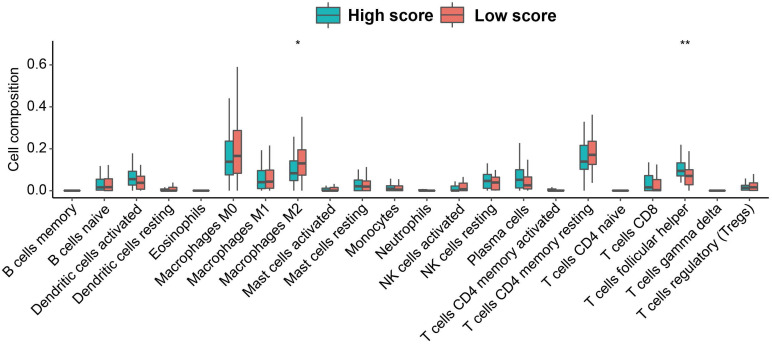
The abundance of 22 immune cell subpopulations in the low and high score group based on the TCGA database.

Through CIBERSORT algorithm, we assessed the relative abundance of the 22 immune cell subpopulations. We found that the low-score group contained significantly higher proportion of Macrophages M2, and lower proportion of T cells follicular helper (Tfh cells) (Figure 6). Additionally, the expression of ATP6V0A1 positively related to the infiltration of B cells. The expression of SLC39A14 related to the infiltration of B cells positively and Dendritic cells negatively. The expression of SLC39A8 positively related to the infiltration of B cells, CD8+ T cells and Macrophages. The expression of SLC3A2 related to the infiltration of B cells, CD8+ T cells, and Neutrophils negatively. The expression of LCN2 related to the infiltration of B cells positively and Macrophages and Dendritic cells negatively. And the expression of PRNP related to the infiltration of B cells and CD4+ T cells negatively and Dendritic cells positively (Figure S7).

### Screening of potential anti-cancer drugs and compounds

**Figure 7:**
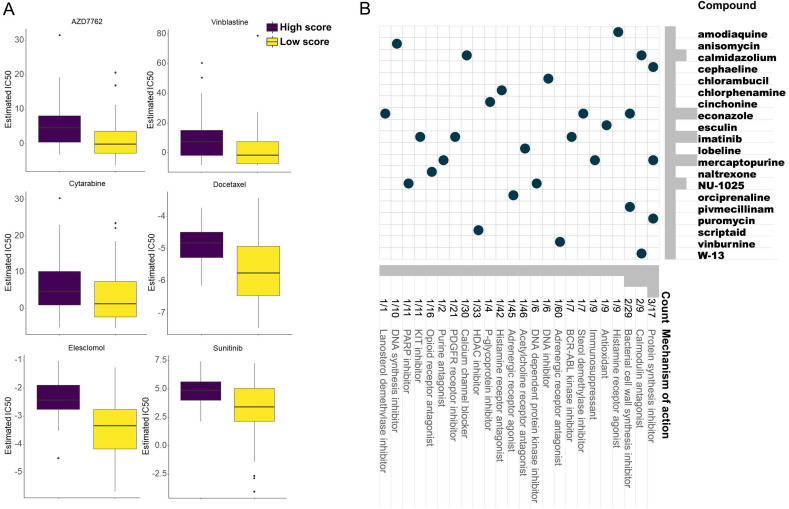
Anti-cancer drugs with higher sensitivity in the low score group based on the GDSC database (A) and potential inhibitors targeting the ESCC prognostic signature based on the CMap database (B).

From the GDSC database, some anti-cancer drugs showed different IC50 between two groups (Figure S8). Of note, AZD7762, Sunitinib, Cytarabine, Docetaxel, Vinblastine, and Elesclomol exhibited lower IC50 in the low-score group, which implied their potential improvement of clinical outcome (Figure 7A).

Three hundred top different expressed genes (150 up-regulated and 150 down-regulated genes) between low- and high-score group were queried in the CMap database to analyze their mechanism of action (MoA) and drug targets. Eventually, 20 potential compounds were identified. In the CMap mode, 24 MoAs were associated with the above compounds. Among them, 3 compounds (cephaeline, mercaptopurine, and puromycin) shared the MoA of protein synthesis inhibitor, 2 compounds (calmidazolium, and W-13) shared the MoA of calmodulin antagonist, and 2 compounds (econazole, and pivmecillinam) shared the MoA of bacterial cell wall synthesis inhibitor. Besides, the antioxidant, DNA synthesis inhibitor, PARP inhibitor, and immunosuppressant were also identified (Figure 7B).

## Discussion

In this study, we identified 6 prognostic ferroptosis-related genes, based on which a prognostic signature was established. The low-score group exhibited a worse OS, which was associated with cancer cell proliferation and migration. Besides, the low-score group had higher CNV and TMB. The P53*,* MUC16*,* and NOTCH1 showed high mutated rates in the low-score group. CIBERSORT algorithm reveal that the low-score group contained significantly higher proportion of Macrophages M2, and lower proportion of T cells follicular helper. In drug analysis, AZD7762, Sunitinib, Cytarabine, Docetaxel, Vinblastine, and Elesclomol exhibited lower IC50 in the low-score group. And a nomogram included the prognostic signature was constructed and validated.

Since first introduced in 2012, more and more researches on ferroptosis have been published, even in plants, protozoa and fungi 20-22. As a new iron-dependent form of regulated cell death, ferroptosis was morphologically characterized by shrunken mitochondria, increased mitochondrial membrane density, and diminished mitochondrial cristae, which cannot be blocked by inhibitors for other regulated cell death, such as apoptosis, autophagy and necroptosis 3. Its potential roles in tumorigenesis, tumor development, and cancer therapy have been widely investigated.

The somatic mutations in the PRNP gene may lead to human prion diseases with fatal and irreversible spongiform generation and astrocytosis in brain lesions 23. Rachidi et al reported a lower lipid peroxidation in in PRNP-transfected neuroblastoma and epithelial cells 24, which was consistent with our findings. The prion protein encoded by the PRNP gene was reported to promote the cancer proliferation of gastric cancer, pancreatic ductal adenocarcinoma, colorectal cancer, glioblastoma, and schwannanoma 25-32. Le Corre et al found that elevated expression of PRNP was associated with a worse OS and relapse-free survival 33. Similar to their results, we also noticed that PRNP was up-regulated in ESCC with a worse OS. System x_c_^-^ is composed of a light-chain subunit (xCT, also known as SLC7A11) and a heavy-chain subunit (CD98hc, also known as SLC3A2), which is responsible for cystine/glutamate exchange and play a vital role in ferroptosis. Digomann et al demonstrated that SLC3A2 knock-out led to reduced GSH level and induction of oxidative stress in HNSCC cells 34. Moreover, several researches reported that SLC3A2 was highly expressed in gastric cancer, osteosarcoma, renal cell carcinoma, and biliary tract cancer 35-38. It has been revealed that high SLC3A2 protein expression meant poor breast cancer-specific survival and distant metastasis-free survival 39. SLC39A8 and SLC39A14 belongs to the ZIP family, which plays an important role in zinc transport and metal-ion import 40-42. Ding et al reported a worse OS in gastric cancer patients with high SLC39A8 expression 43. SLC39A14 was also reported to be up-regulated, which implied a better OS in gastric cancer patients 43. However, in our study, the high expression of SLC39A14 in ESCC meant a worse OS. As we know, up-regulated genes in malignancies were more likely to be oncogenic and unfavorable for survival. The role of SLC39A14 in ESCC needs to be further investigated in large-scale researches. ATP6V0A1 is a member of the vacuolar H^+^-ATPases, which were multimeric proton pumps and involved in acidify various intra-cellular organelles. Gleize et al reported that ATP6V0A1 was highly expression in grade III oligodendrogliomas and oligoastrocytomas with a shorter OS 44. In the contrary, we found that ATP6V0A1 was down-regulated in ESCC with a worse OS. LCN2 is a secreted glycoprotein of the adipokine superfamily, which maintains the iron homeostasis 45. Xiao et al revealed the facilitation of LCN2 in tumor iron-uptake 46. We also observed the facilitation of LCN2 in ferroptosis. Maier et al revealed that LCN2 was overexpressed in the colorectal carcinoma with a poor OS. Its high expression in high-grade endometrial cancer was also reported, which related to the shorter OS and disease-free survival. However, Monisha et al exhibited that LCN2 was down-regulated in oral cancer. Furthermore, knock-down of LCN2 led to increased viability of oral cancer cells 47. Similarly, we also observed the lower expression of LCN2 in ESCC with a worse OS.

Somatic mutations may generate tumor-specific neoantigens which play a critical role in the response to immune checkpoint inhibitors (ICIs). The great response of non-small cell lung cancer and melanoma with high TMB to ICIs was observed with improved survival 48. Galsky et al reported that TMB combined with PD-L1 better predicted objective response rate (ORR), progression-free survival (PFS), and OS than PD-L1 alone 49. Hanna et al also demonstrated that higher TMB among virus-negative squamous cell carcinoma of the head and neck tumors predicted anti-PD-1/L1 response 50. It has been reported that tumor-associated macrophages M2 secreted several immunosuppressive molecules to attenuate the responses of T cells to the tumor antigens 51. Sugimura et al exhibited that high infiltration of macrophages M2 was associated with a poor response to chemotherapy and poor prognosis in ESCC patients after surgery, which was consistent with our results 52. A few researches revealed that Tfh cells help the development of ectopic lymphoid structures, which were involved in the antitumor immunity 53, 54. The specific role of Tfh cells in malignancies is still unclear and further investigation is necessary.

The GDSC database characterized 1000 human cancer cell lines and screened them with more than 100 anti-cancer drugs. It systematically integrated large-scale genomic alterations and drug sensitivity datasets in cancer 18. As we know, the development of new drugs for specific malignancies was lengthy and tortuous and thousands of failures were inevitable. The existing drugs have passed safety assessment, which means the investigation of their uses for other cancers can be time-saving and economical. We identified some anti-cancer drugs with lower IC50 in the low-score group based on the GDSC database, which may be helpful for the discovery of drugs for ESCC.

It should be noted that there are several imitations of our study. First, limited number of ESCC samples provided clinical outcome. Large-scale researches across multiple areas are needed to explore the detailed association of ferroptosis with the prognosis of ESCC. Second, links between the prognostic score and immune infiltration and the compounds were not experimentally validated.

## Conclusion

We identified 6 ferroptosis- and iron metabolism-related prognostic genes in ESCC, based on which a prognostic signature was constructed and validated. A nomogram including age, grade, T stage, N stage, TNM stage, adjuvant therapy, and prognostic signature was also established and validated to accurately predict the OS of ESCC patients. The potential mechanisms between ferroptosis-related genes and tumor immunity and drug sensitivity still warrant further studies.

## Supplementary Material

Supplementary figures and table 1.

Supplementary table 2.

## Figures and Tables

**Table 1:** Characteristics of 112 ESCC patients from Zhongshan Hospital, Fudan University

	Number	Percent (%)
**Age (years)**		
<55	27	24.1
55-65	51	45.5
>65	34	30.4
**Sex**		
Male	86	76.8
Female	26	23.2
**Smoke**		
Yes	64	57.1
No	48	42.9
**Stage**		
0	2	1.8
I	38	33.9
II	30	26.8
III	39	34.8
IV	3	2.7
**Fluorescence intensity (Mean)**		
PRNP	55.5	
SLC3A2	52.9	
SLC39A8	48.4	
LC39A14	47.6	
ATP6V0A4	34.5	
LCN2	39.9	

## Abbreviations

ESCC: esophageal squamous cell carcinoma
EAC: esophageal adenocarcinoma
GSH: glutathione
CNV: copy number variation
GEO database: Gene Expression Omnibus database
TCGA database: The Cancer Genome Atlas database
GDSC database: Genomics of Drug Sensitivity in Cancer database
CMap database: Connectivity Map database
DEGs: differentially expressed genes
ROC curve: time-dependent receptor operating characteristic curves
GO/KEGG analysis: Gene Ontology and Kyoto Encyclopedia of Genes and Genomes analysis
OS: overall survival
C-index: Harrel concordance index
AUC: area under the curve
Tfh cells: T cells follicular helper
MoA: mechanism of action
ICIs: immune checkpoint inhibitors
ORR: objective response rate
PFS: progression-free survival
