# Supplementary material for: Identification and analysis of a prognostic ferroptosis and iron-metabolism signature for esophageal squamous cell carcinoma
Source: J Cancer. 2022 Mar 6;13(5):1611–22. doi: 10.7150/jca.68568 (PMC8965120; doi:10.7150/jca.68568)
Supplement: Supplementary file 1 — Supplementary figures and table 1. [file jcav13p1611s1.pdf]

Figure S1. Heatmap of differentially expressed genes between ESCC and normal esophageal tissues from the GSE20347, GSE67269, and GSE38129 datasets.

Figure S2: Lipid peroxidation after transfection with siRNAs in ECA109 cells.

Figure S3: GSEA analysis of the prognostic signature.

Figure S4: Kaplan-Meier curves of grade (A), T stage (B), stage (C), age (D), adjuvant therapy (E), and N stage (F) from the GSE53625 dataset.

Figure S5: Forest map of the 112 ESCC patients from Zhongshan Hospital, Fudan University.

Figure S6: Correlation of highly mutated genes in the low and high score group based on the TCGA database.

Figure S7: Correlation of key genes expressions and immune cell infiltration based on the TIMER database.

Figure S8: Anti-cancer drug sensitivity between the low and high score group based on the GDSC database.

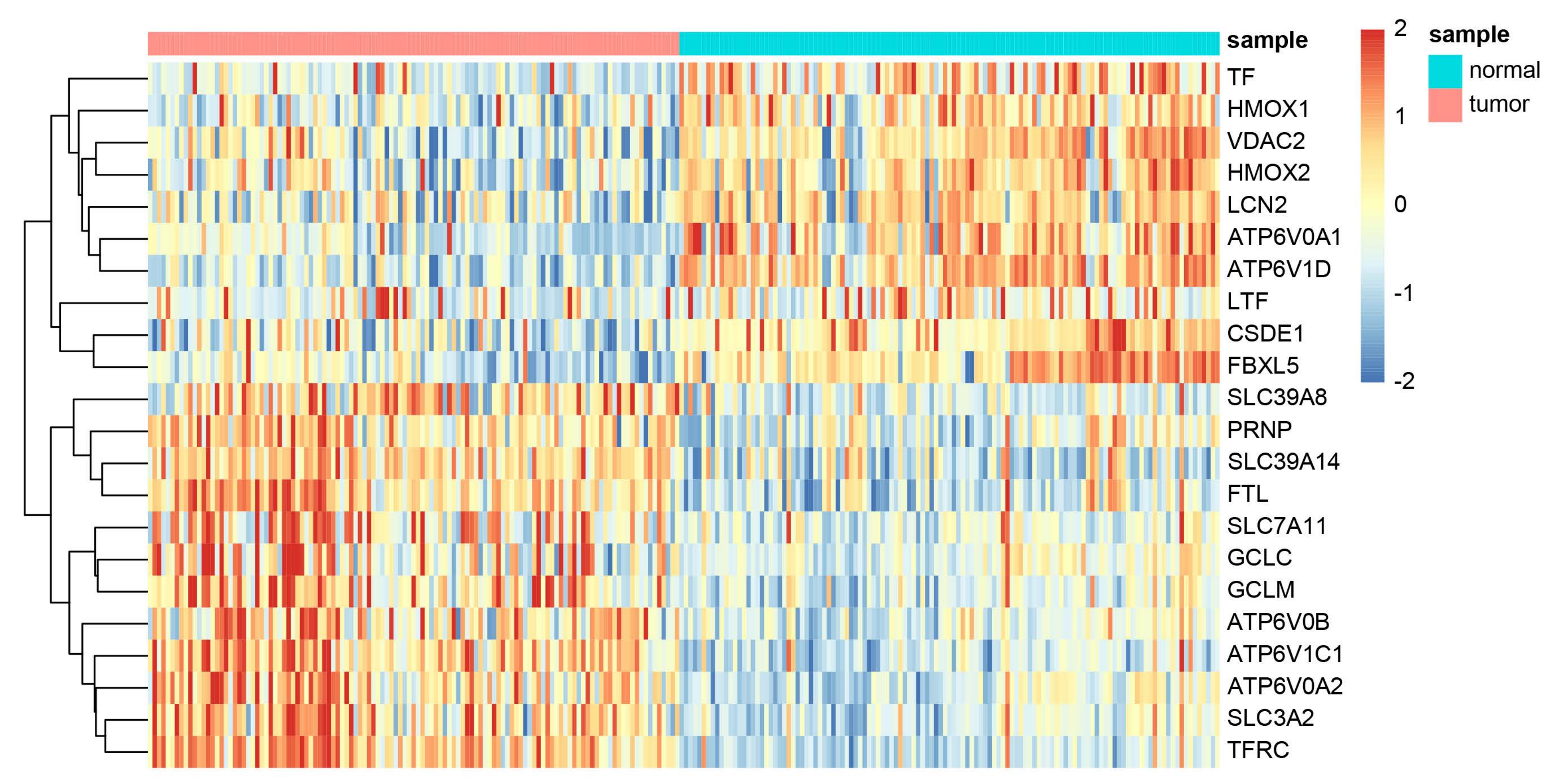
sample
TF
HMOX1
VDAC2
HMOX2
LCN2
ATP6V0A1
ATP6V1D
LTF
CSDE1
FBXL5
SLC39A8
PRNP
SLC39A14
FTL
SLC7A11
GCLC
GCLM
ATP6V0B
ATP6V1C1
ATP6V0A2
SLC3A2
TFRC
2
1
0
-1
-2
sample
normal
tumor

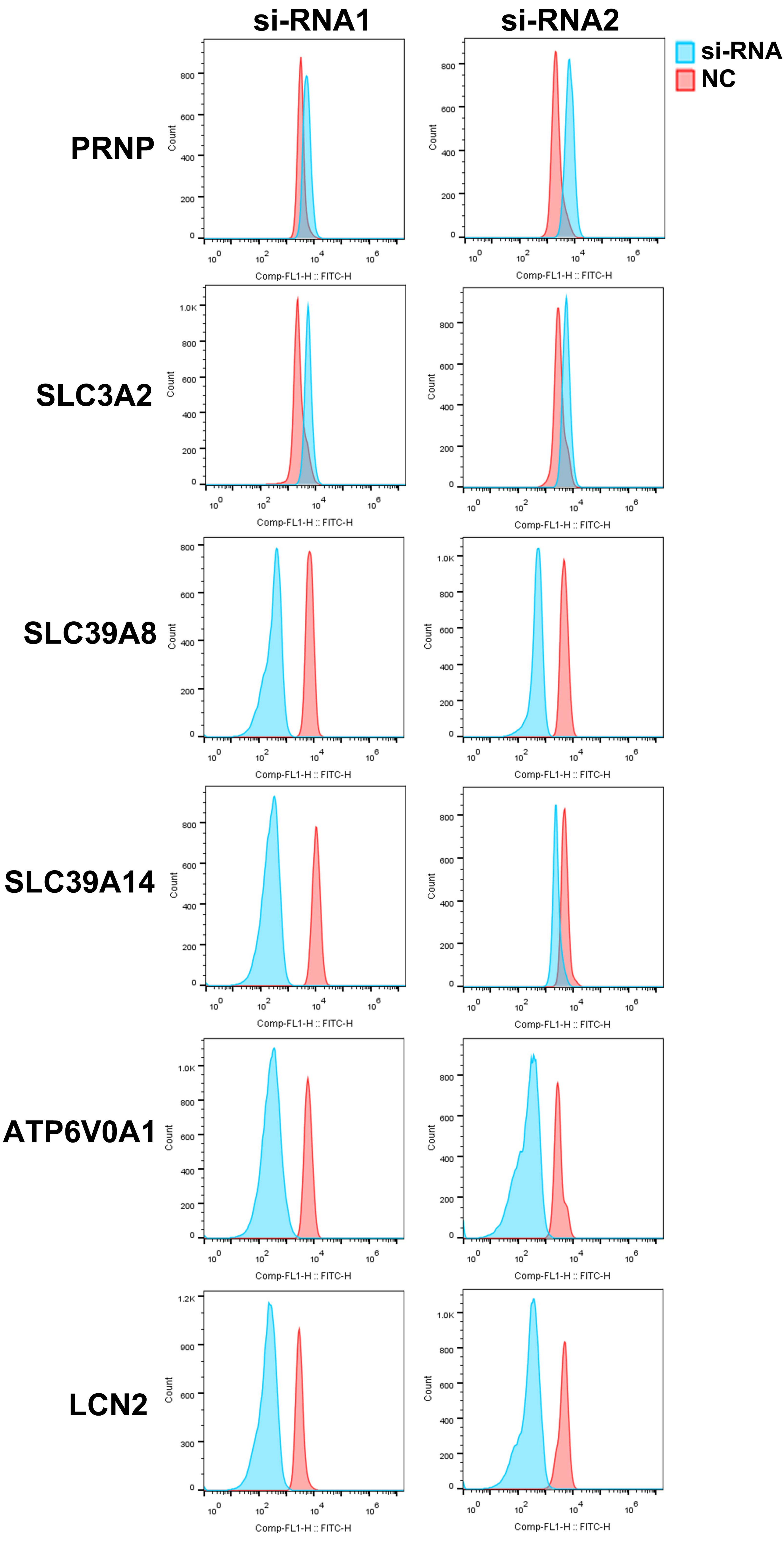
si-RNA1
si-RNA2
si-RNA
NC
PRNP
SLC3A2
SLC39A8
SLC39A14
ATP6V0A1
LCN2
Count
Comp-FL1-H :: FITC-H

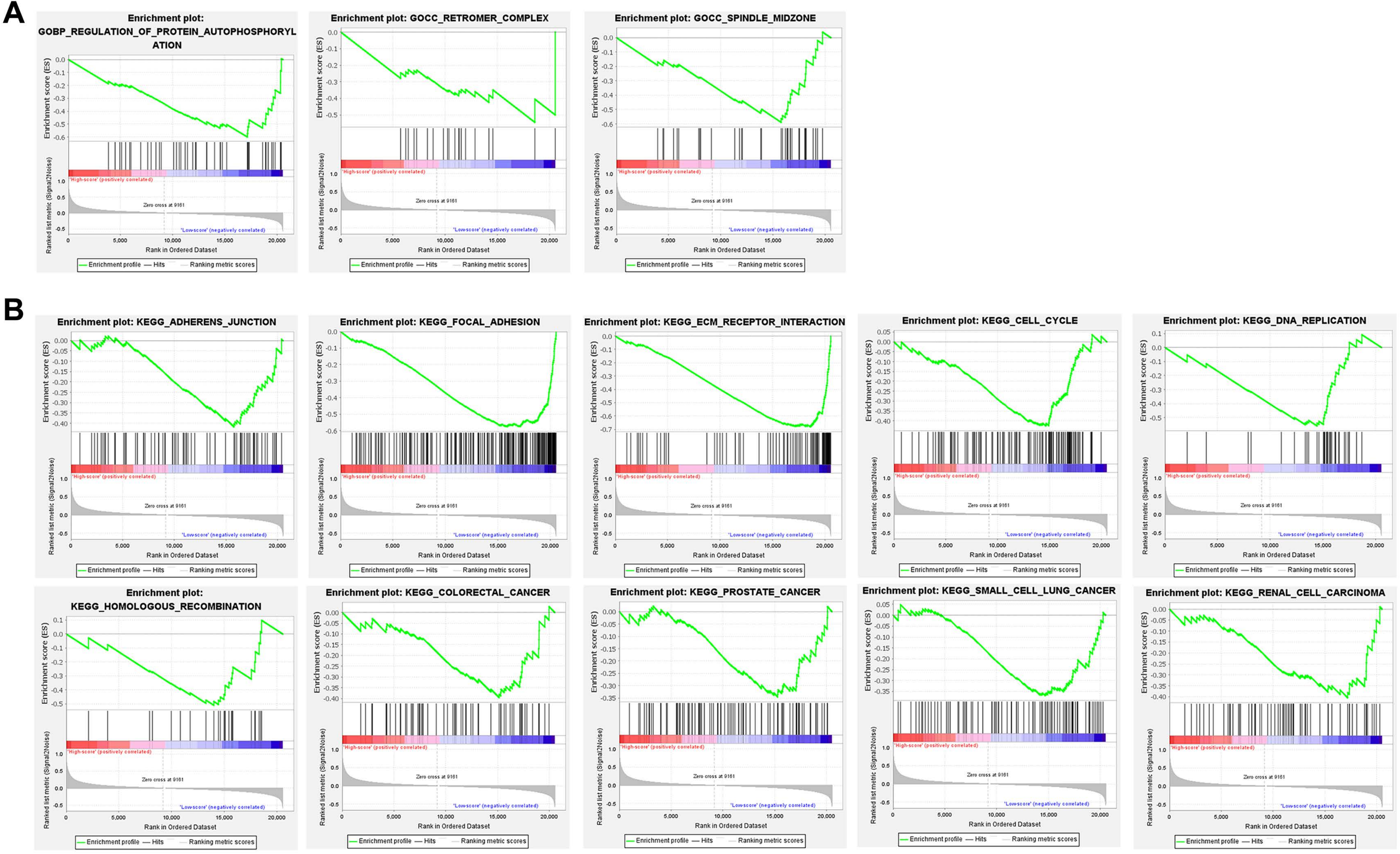
A
Enrichment plot: GOBP_REGULATION_OF_PROTEIN_AUTOPHOSPHORYLATION
Enrichment plot: GOCC_RETROMER_COMPLEX
Enrichment plot: GOCC_SPINDLE_MIDZONE
B
Enrichment plot: KEGG_ADHERENS_JUNCTION
Enrichment plot: KEGG_FOCAL_ADHESION
Enrichment plot: KEGG_ECM_RECEPTOR_INTERACTION
Enrichment plot: KEGG_CELL_CYCLE
Enrichment plot: KEGG_DNA_REPLICATION
Enrichment plot: KEGG_HOMOLOGOUS_RECOMBINATION
Enrichment plot: KEGG_COLORECTAL_CANCER
Enrichment plot: KEGG_PROSTATE_CANCER
Enrichment plot: KEGG_SMALL_CELL_LUNG_CANCER
Enrichment plot: KEGG_RENAL_CELL_CARCINOMA
Enrichment score (ES)
Ranked list metric (Signal2Noise)
'High-score' (positively correlated)
Zero cross at 9161
'Low-score' (negatively correlated)
Rank in Ordered Dataset
Enrichment profile — Hits Ranking metric scores

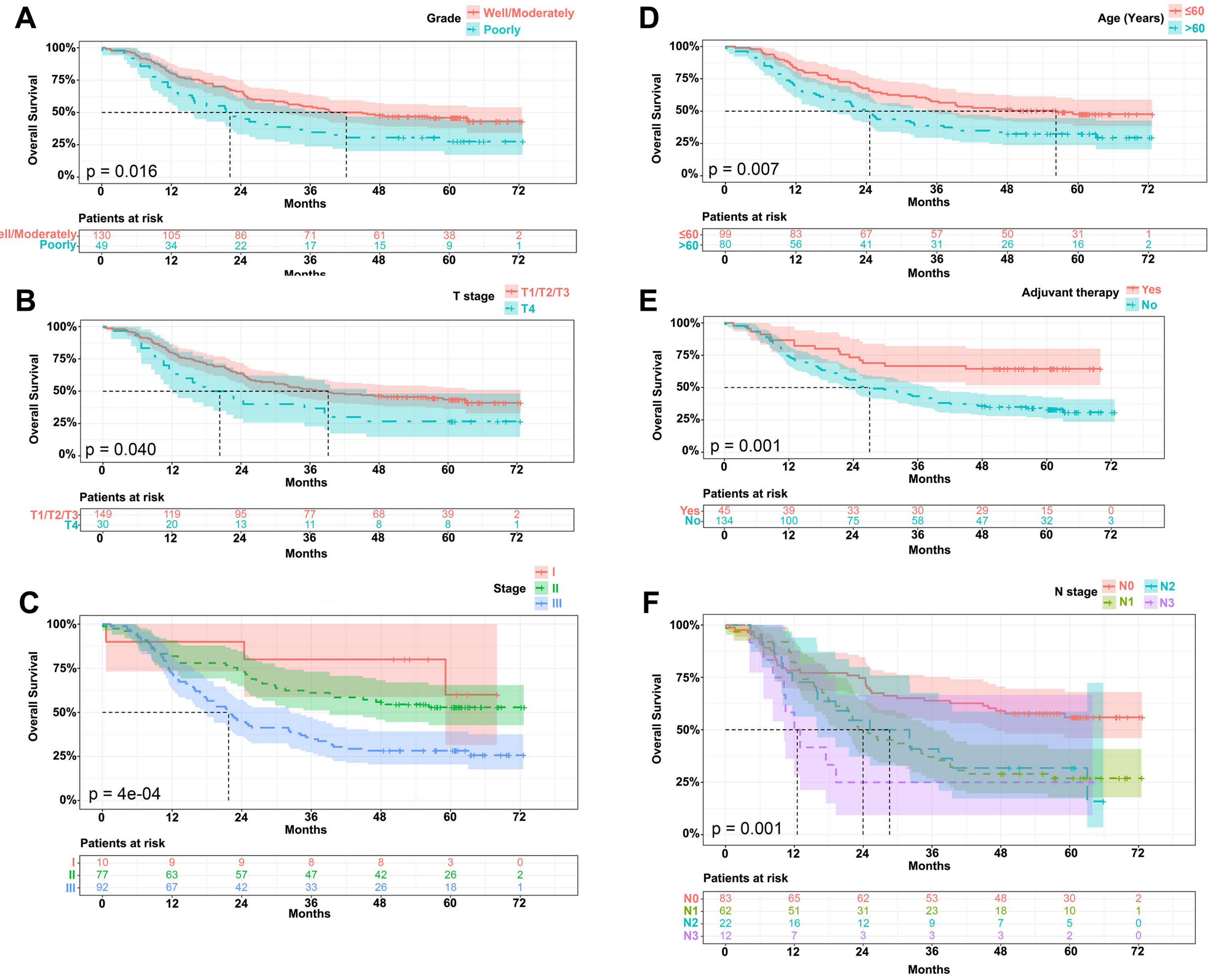

A
Grade
Well/Moderately
Poorly
Overall Survival
p = 0.016
Months
Patients at risk
Well/Moderately 130 105 86 71 61 38 2
Poorly 49 34 22 17 15 9 1
B
T stage
T1/T2/T3
T4
p = 0.040
T1/T2/T3 149 119 95 77 68 39 2
T4 30 20 13 11 8 8 1
C
Stage
I
II
III
p = 4e-04
I 10 9 9 8 8 3 0
II 77 63 57 47 42 26 2
III 92 67 42 33 26 18 1
D
Age (Years)
≤60
>60
p = 0.007
≤60 99 83 67 57 50 31 1
>60 80 56 41 31 26 16 2
E
Adjuvant therapy
Yes
No
p = 0.001
Yes 45 39 33 30 29 15 0
No 134 100 75 58 47 32 3
F
N stage
N0
N1
N2
N3
p = 0.001
N0 83 65 62 53 48 30 2
N1 62 51 31 23 18 10 1
N2 22 16 12 9 7 5 0
N3 12 7 3 3 3 2 0

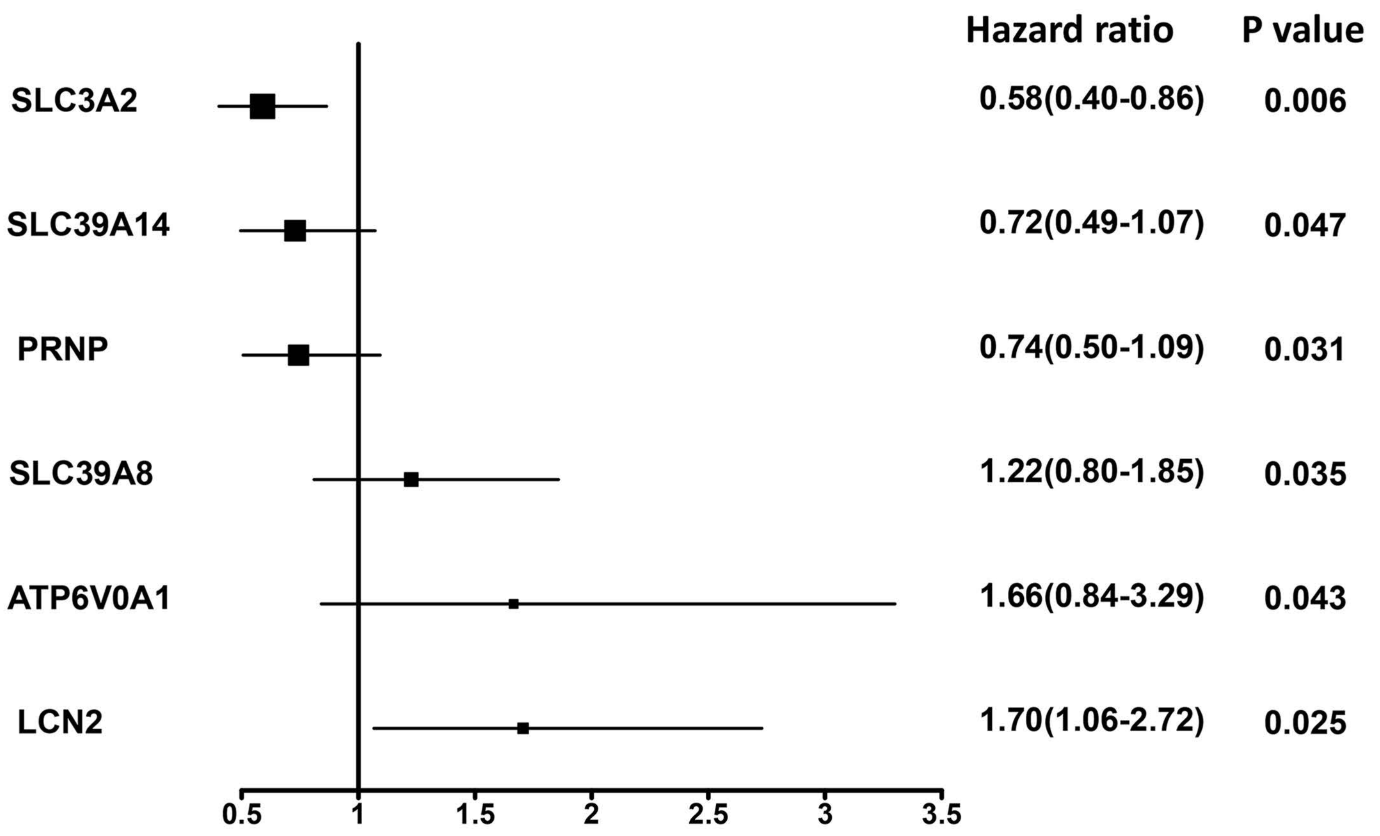
Hazard ratio
P value
SLC3A2
0.58(0.40-0.86)
0.006
SLC39A14
0.72(0.49-1.07)
0.047
PRNP
0.74(0.50-1.09)
0.031
SLC39A8
1.22(0.80-1.85)
0.035
ATP6V0A1
1.66(0.84-3.29)
0.043
LCN2
1.70(1.06-2.72)
0.025
0.5
1
1.5
2
2.5
3
3.5

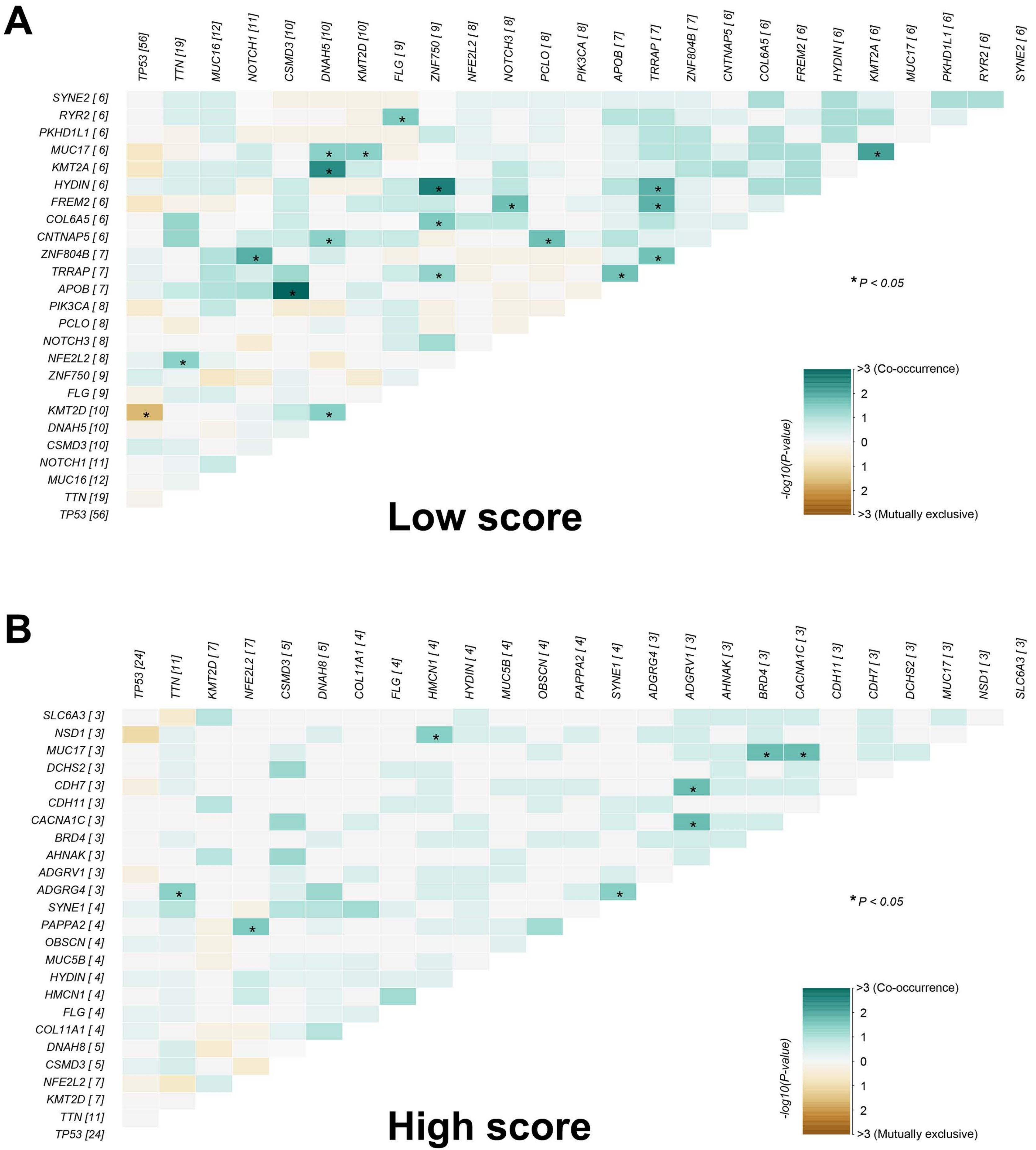
A
TP53 [56]
TTN [19]
MUC16 [12]
NOTCH1 [11]
CSMD3 [10]
DNAH5 [10]
KMT2D [10]
FLG [9]
ZNF750 [9]
NFE2L2 [8]
NOTCH3 [8]
PCLO [8]
PIK3CA [8]
APOB [7]
TRRAP [7]
ZNF804B [7]
CNTNAP5 [6]
COL6A5 [6]
FREM2 [6]
HYDIN [6]
KMT2A [6]
MUC17 [6]
PKHD1L1 [6]
RYR2 [6]
SYNE2 [6]
* P < 0.05
>3 (Co-occurrence)
2
1
0
1
2
>3 (Mutually exclusive)
-log10(P-value)
Low score
B
TP53 [24]
TTN [11]
KMT2D [7]
NFE2L2 [7]
CSMD3 [5]
DNAH8 [5]
COL11A1 [4]
FLG [4]
HMCN1 [4]
HYDIN [4]
MUC5B [4]
OBSCN [4]
PAPPA2 [4]
SYNE1 [4]
ADGRG4 [3]
ADGRV1 [3]
AHNAK [3]
BRD4 [3]
CACNA1C [3]
CDH11 [3]
CDH7 [3]
DCHS2 [3]
MUC17 [3]
NSD1 [3]
SLC6A3 [3]
* P < 0.05
>3 (Co-occurrence)
2
1
0
1
2
>3 (Mutually exclusive)
-log10(P-value)
High score

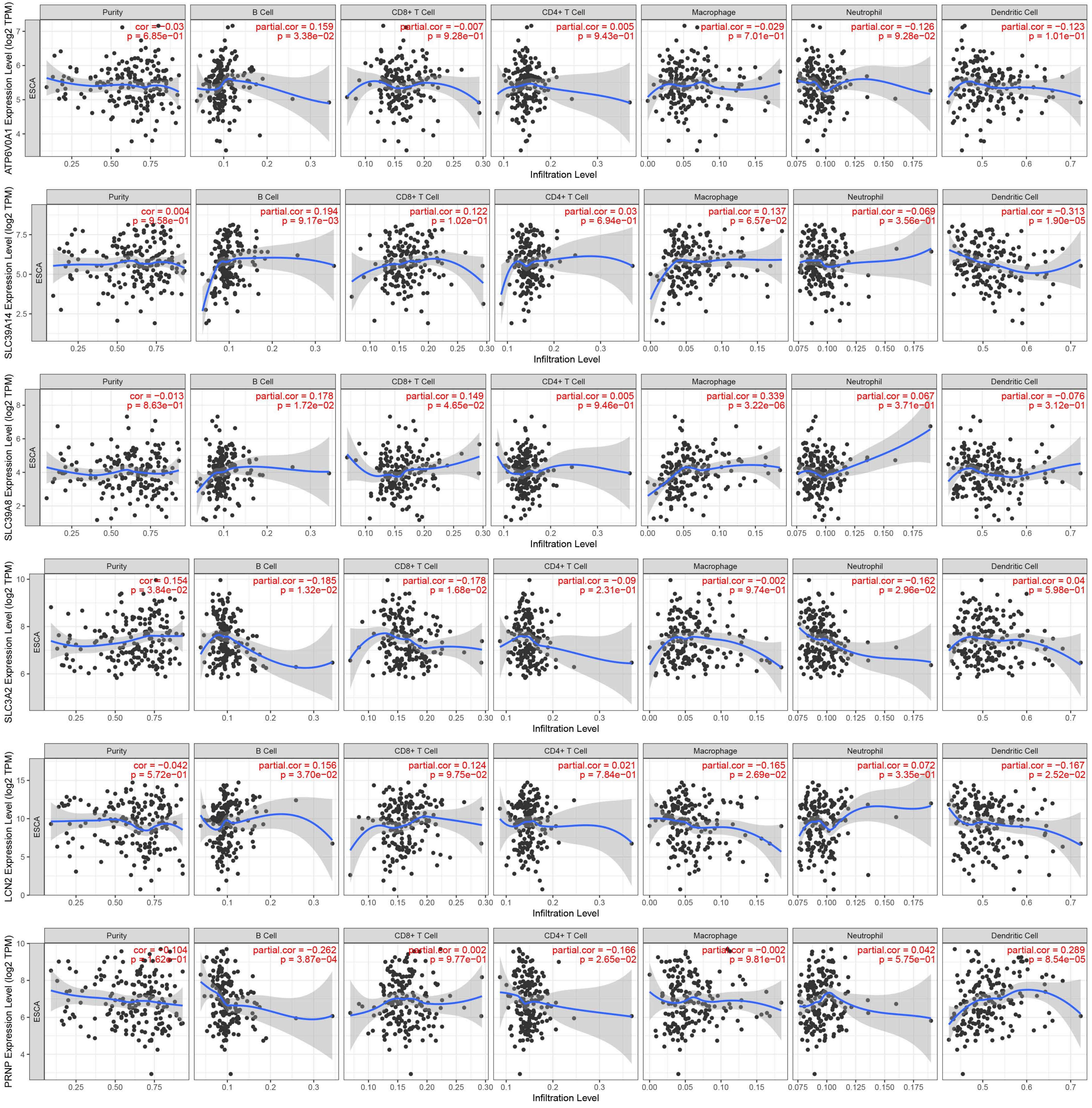

Purity
B Cell
CD8+ T Cell
CD4+ T Cell
Macrophage
Neutrophil
Dendritic Cell
ESCA
ATP6V0A1 Expression Level (log2 TPM)
cor = −0.03
p = 6.85e−01
partial.cor = 0.159
p = 3.38e−02
partial.cor = −0.007
p = 9.28e−01
partial.cor = 0.005
p = 9.43e−01
partial.cor = −0.029
p = 7.01e−01
partial.cor = −0.126
p = 9.28e−02
partial.cor = −0.123
p = 1.01e−01
SLC39A14 Expression Level (log2 TPM)
cor = 0.004
p = 9.58e−01
partial.cor = 0.194
p = 9.17e−03
partial.cor = 0.122
p = 1.02e−01
partial.cor = 0.03
p = 6.94e−01
partial.cor = 0.137
p = 6.57e−02
partial.cor = −0.069
p = 3.56e−01
partial.cor = −0.313
p = 1.90e−05
SLC39A8 Expression Level (log2 TPM)
cor = −0.013
p = 8.63e−01
partial.cor = 0.178
p = 1.72e−02
partial.cor = 0.149
p = 4.65e−02
partial.cor = 0.005
p = 9.46e−01
partial.cor = 0.339
p = 3.22e−06
partial.cor = 0.067
p = 3.71e−01
partial.cor = −0.076
p = 3.12e−01
SLC3A2 Expression Level (log2 TPM)
cor = 0.154
p = 3.84e−02
partial.cor = −0.185
p = 1.32e−02
partial.cor = −0.178
p = 1.68e−02
partial.cor = −0.09
p = 2.31e−01
partial.cor = −0.002
p = 9.74e−01
partial.cor = −0.162
p = 2.96e−02
partial.cor = 0.04
p = 5.98e−01
LCN2 Expression Level (log2 TPM)
cor = −0.042
p = 5.72e−01
partial.cor = 0.156
p = 3.70e−02
partial.cor = 0.124
p = 9.75e−02
partial.cor = 0.021
p = 7.84e−01
partial.cor = −0.165
p = 2.69e−02
partial.cor = 0.072
p = 3.35e−01
partial.cor = −0.167
p = 2.52e−02
PRNP Expression Level (log2 TPM)
cor = −0.104
p = 1.62e−01
partial.cor = −0.262
p = 3.87e−04
partial.cor = 0.002
p = 9.77e−01
partial.cor = −0.166
p = 2.65e−02
partial.cor = −0.002
p = 9.81e−01
partial.cor = 0.042
p = 5.75e−01
partial.cor = 0.289
p = 8.54e−05
Infiltration Level

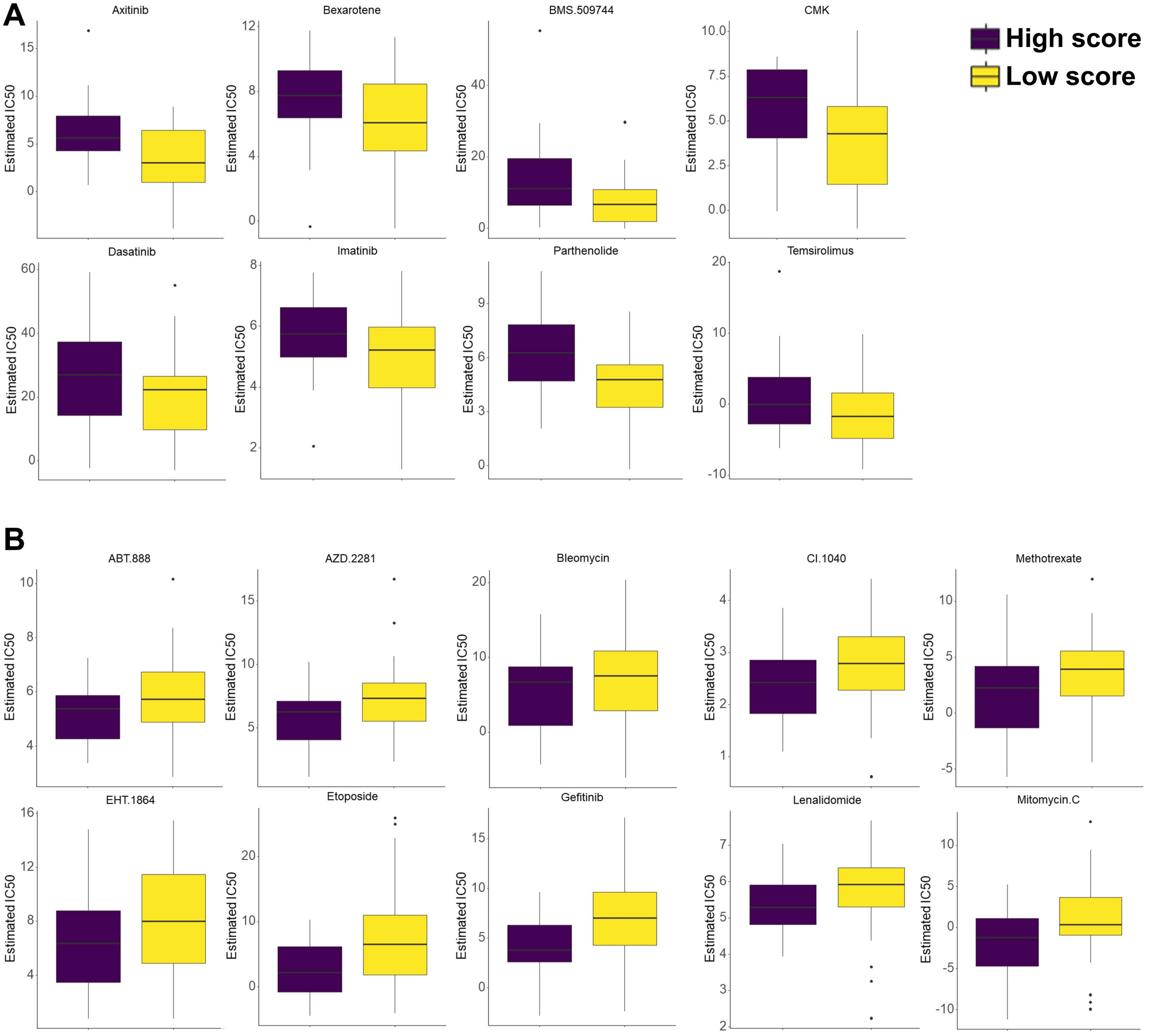

A
B
High score
Low score
Axitinib
Bexarotene
BMS.509744
CMK
Dasatinib
Imatinib
Parthenolide
Temsirolimus
ABT.888
AZD.2281
Bleomycin
CI.1040
Methotrexate
EHT.1864
Etoposide
Gefitinib
Lenalidomide
Mitomycin.C
Estimated IC50

Table S1: Sequence of siRNAs and Primers of prognostic genes.

	siRNA1 (5’-3’)	siRNA2 (5’-3’)	Primer (5’-3’)
PRNP	AUCAUUACAGCAGUGAAACUG	AAACACUUCAAAUCAUAUGGG	F: AGTCAGTGGAACAAGCCGAG<br>R: CTGCCGAAATGTATGATGGGC
SLC3A2	UAGAUGUUUACCACAAACGAC	AUCUUUCAUGAAUUGUAGCUA	F: TGAATGAGTTAGAGCCCGAGA<br>R: GTCTTCCGCCACCTTGATCTT
SLC39A8	UCAUAUACAAAGUGAAGUCUA	UAAGUAACUCAUUCCUAUCUA	F: ATGCTACCCAAATAACCAGCTC<br>R: ACAGGAATCCATATCCCCAAACT
SLC39A14	UGGUUAAGGAGAUCUAGUCUG	UUUCUUCUCUGAAGAAAUCAG	F: GAGGCTCACGCTTCATCCC<br>R: CCCTCGCCATACCGATGTATTA
ATP6V0A1	UGAAGAAACAGCAAUUCUGCA	UAUGAAGAAACAGCAAUUCUG	F: GGGAGCGCATCCCTACTTTT<br>R: GGTTCTCGATTTCAGCCTGTC
LCN2	UGACAUUGUAGCUCUUGUCUU	UGUGCUAUAAACGUUGCUCUA	F: CCACCTCAGACCTGATCCCA<br>R: CCCCTGGAATTGGTTGTCCTG
